# Advantages and Disadvantages of Electronic Cigarettes

**DOI:** 10.3390/toxics11010066

**Published:** 2023-01-11

**Authors:** Andrzej Sobczak, Leon Kośmider

**Affiliations:** Department of General and Inorganic Chemistry, Faculty of Pharmaceutical Sciences in Sosnowiec, Medical University of Silesia, 40-055 Katowice, Poland

Despite nearly nine thousand publications on e-cigarettes (EC) in the PubMed database, there is still no consensus in the scientific community and among decision makers regarding the risks and benefits of using these products. As we emphasized in the call for papers, further research is needed to provide new evidence-based knowledge to better inform the public about the possible risks as well as the benefits for smokers related to the use of e-cigarettes. We proposed a wide range of topics, which included laboratory studies related to the presence of harmful substances in the liquid and aerosol, in vivo and in vitro health effects studies, the role of nicotine in addiction, and observational population studies on the use of EC.

The papers submitted for the Special Issue (SI) fit into the proposed topics. Two papers concern reactive oxygen species (ROS) generated during the use of an e-cigarette containing synthetic nicotine [1] and the influence of flavoring substances on the appearance of ROS in the aerosol [2]. Flavoring substances are also the subject of research by Bebenek et al. [3]. The authors analyze their influence on the content of free and protonated nicotine and the consequences associated with nicotine addiction. In turn, animal studies [4] have hypothesized that exposure to flavored e-cigarettes would cause lung inflammation in C57BL/6 J mice. This study revealed that flavor-based e-cigarette exposure elicited sex-specific alterations in lung inflammation, with cherry flavors/benzaldehyde eliciting female-specific and tobacco flavor resulting in male-specific increases in lung inflammation. Such studies indicate the potential toxicity of some flavorings added to e-liquid which should be taken into account when formulating regulations.

In in vivo studies, Cichońska et al. [5] conclude that e-cigarette usage adversely affects the antioxidant capacity of saliva, in comparison to non-smokers, to the same extent as smoking traditional cigarettes. This might present an important clinical risk of oral cavity disorders. Additionally, in their review paper, Szumilas et al. [6] review the literature in terms of the impact of e-cigarette aerosol on the cells and tissues of the oral cavity.

In turn, in vitro studies have shown that e-cigarette vapor condensate (ECVC) has a negative effect on both osteoblast viability and function, with these effects being mediated, in part, by nicotine-dependent mechanisms and also reactive carbonyl species derived from e-liquid humectants. Reduced osteoblast viability, coupled with a reduction in OPG secretion as observed following ECVC treatment, may lead to increased bone resorption following chronic exposure, in turn potentially impacting bone development in younger users, while increasing bone-associated disease progression and negatively impacting orthopedic and dental surgery outcomes [7].

Another article in this SI is devoted to the study of the storage conditions and type of clearomizers on the increase in heavy metal levels in e-cigarette liquids retailed in Romania [8]. It has been found that the long period and high storage temperature of e-liquids in the clearomizer have an effect on increasing the level of heavy metals in the generated aerosol. This is important information for users of these products, aiming to reduce the harmfulness of their use.

In many reports published by prestigious scientific institutions, special attention is paid to the threat that e-cigarettes may pose to young people. Therefore, we welcomed the paper describing the results of a cross-sectional study conducted in Poland [9]. The main aim of this study was the assessment of the factors associated with the use of electronic cigarettes among high school students. Two parameters used to assess public health were used for this purpose: health literacy (HL) and the health locus of control (HLC). Personal health literacy is the degree to which individuals have the ability to find, understand, and use information and services to inform health-related decisions and actions for themselves and others. The health locus of control refers to the belief that health is in one’s control (internal control) or is not in one’s control (external control). Among adults, the external locus of control is associated with negative health outcomes, whereas the internal locus of control is associated with favorable outcomes. The obtained results showed that students smoking conventional cigarettes were more prone to using e-cigarettes. To sum up, it was an unexpected result that HL is not associated with the use of e-cigarettes. A greater likelihood of using e-cigarettes was positively associated with higher HLC scores, as in the case of traditional smoking.

There are currently ongoing debates about the relationship between e-cigarette use, NRT use, and the uptake and provision of other quit methods including behavioral support. It has been suggested, for instance, that widespread e-cigarette use may be reducing the need for stop smoking services (SSSs). Meanwhile, research by Harweell et al. [10] does not support this argument; some smokers participating in the study were still willing to receive additional support in quitting from SSSs, even if they were already using e-cigarettes.

Another paper [11] uses data from Wave 3 of The Population Assessment of Tobacco and Health (PATH) study which is a nationally representative longitudinal study of tobacco use and health in the United States. The authors assess associations between e-cigarette use and self-reported hypertension, a highly prevalent health condition and major contributor to cardiovascular disease burden. According to the authors, after adjusting for potential confounders, current vaping (OR = 1.31) and current smoking (OR = 1.27) were both associated with higher odds of hypertension; those odds were lower for respondents who were concurrently smoking and vaping (OR = 1.77). The results obtained make an important contribution to the evaluation of the association of e-cigarette use with major adverse cardiovascular endpoints (e.g., stroke and myocardial infarction).

Controversies around the risks posed by e-cigarettes are often due to the wide variety of products and user behavior, the underestimation or overestimation of risk, as well as the wrong methodological approach. In this context, we pay particular attention to two further works. Talhout et al. [12] used several approaches to quantify the health risk of tobacco products, either the absolute risk or that relative to a tobacco cigarette. The hazard index (HI) and relative potency factor (RPF) approaches may be used for the quantification of health risk, provided that sufficient and relevant hazard and exposure data are available. None of the methods are ready to be used in regulation yet due to a lack of relevant data on hazard and exposure, but also due to a variety of regulatory needs and wishes. However, the application of these methods may be possible in due time.

One of the reasons for the controversy surrounding e-cigarettes is the different, often contradictory results of studies covering the same research topic. The reasons may vary. However, the most important is the research methodology. This topic was discussed in two papers by Soulet and Sussman. In the first paper [13], the authors critically reviewed laboratory studies published after 2017 on the metal content of EC aerosol, focusing on the consistency between their experimental design, the actual use of the device, and the corresponding exposure risk assessment. The authors showed the most important reasons for the variation in results in the reviewed papers. They included inadequate BA test protocols unsuited to the power of the heater; miscalculation of exposure levels based on experimental results; devices manufactured many months before the experiment, which could be the cause of corrosion of the e-cigarette’s metal components; and lack of sufficient information to allow repetition of the study.

Similar topics are addressed in the second paper [14]. They review the literature on laboratory studies quantifying the production of potentially toxic organic by-products (carbonyls, carbon monoxide, and free radicals) in e-cigarette aerosol emissions, focusing on the consistency between their experimental design and a realistic usage of the devices. The authors conclude that laboratory testing requires a much more flexible standard, not only providing appropriate technical guidelines, but facilitating the incorporation of end users to complement laboratory logistics.

We agree with the authors of these papers that an objective assessment of the risk of using e-cigarettes requires the elimination of incorrect research methodology and signals the necessity to upgrade current laboratory-testing standards.

The papers posted in the SI cover various research areas related to e-cigarettes. In our opinion, they show two important directions for further research. The first is the role of flavor additives in the overall assessment of the harmfulness of e-cigarettes, and the second is the need to take steps toward standardizing methods at least for areas of research in which we observe considerable variation in the results obtained, which at present makes it difficult to take rational regulatory action and recommendations.
